# Exploitation of Perennial Plant Biomass for Particleboards Designed for Insulation Applications

**DOI:** 10.3390/ma18020352

**Published:** 2025-01-14

**Authors:** Danuta Martyniak, Marta Pędzik, Grzegorz Żurek, Karol Tomczak, Ryszard Gąsiorowski, Magdalena Komorowicz, Dominika Janiszewska-Latterini

**Affiliations:** 1 Plant Breeding and Acclimatization Institute, National Research Institute, Radzików, 05-870 Błonie, Poland; d.martyniak@ihar.edu.pl; 2 Łukasiewicz Research Network—Poznań Institute of Technology, Ewarysta Estkowskiego 6, 61-755 Poznan, Poland; marta.pedzik@pit.lukasiewicz.gov.pl (M.P.); karol.tomczak@pit.lukasiewicz.gov.pl (K.T.); ryszard.gasiorowski@pit.lukasiewicz.gov.pl (R.G.); magdalena.komorowicz@pit.lukasiewicz.gov.pl (M.K.)

**Keywords:** prairie cordgrass, giant miscanthus, switchgrass, tall wheatgrass, cup plant, formaldehyde emissions, thermal conductivity

## Abstract

With rising demand for wood products and reduced wood harvesting due to the European Green Deal, alternative lignocellulosic materials for insulation are necessary. In this work, we manufactured reference particleboard from industrial particles and fifteen different board variants from alternative lignocellulosic plants material, i.e., five types of perennial plant biomass in three substitutions: 30, 50 and 75% of their share in the board with a nominal density of 250 kg/m^3^. Within the analysis of manufactured boards, the mechanical, chemical and thermal properties were investigated—internal bond, formaldehyde emissions, thermal insulation, heat transfer coefficient and thermal conductivity. In the case of thermal conductivity, the most promising results from a practical point of view (W/mK < 0.07) were obtained with *Sida hermaphrodita* and *Miscanthus*, achieving the best results at 50% substitution. The lowest formaldehyde emissions were recorded for boards with *Panicum virgatum* and *Miscanthus*, highlighting their positive environmental performance. In terms of mechanical properties, the highest internal bond was noticed in particleboards with a 30% substitution of *Spartina pectinata* and *Miscanthus*. Research findings confirm the potential of perennial plants as a sustainable source of raw materials for insulation panel manufacturing. Despite needing improvements in mechanical properties, most notably internal bond strength, these plants offer an ecologically responsible solution aligned with global construction trends, thus lessening reliance on traditional wood products. Thus, long-term benefits may be realized through the strategic combination of diverse raw materials within a single particleboard.

## 1. Introduction

Although the Food and Agricultural Organization of the United Nations forecasts higher demand for wooden products in the next decades [1]. The new EU Green Deal (EGD) requires the limiting of forest harvesting and an increase in the cover of protected areas [2] in order to mitigate climate change effects through continuous forest cover and afforestation [3,4].

Nonetheless, wood is a fundamental, sustainable material for construction and building worldwide [5,6,7] when meeting EGD requirements, athough the amount of available good-quality wood may be lower due to several challenges. Therefore, currently one of the biggest challenges of the construction sector is to find alternative raw materials to replace wood [8,9].

Finding non-wood alternatives to large-size timber is a complex task, especially considering the exceptional quality that can be achieved by various tree species, even in challenging environments like post-agricultural lands and re-cultivated areas [10,11]. Nonetheless, several wooden goods have the potential to be substituted with alternative lignocellulosic biomass, either partially or entirely [12,13]. Wood-based panels are included in the list of such products [14]. However, manufacturing composite materials based on lignocellulosic biomass requires maintaining the proper characteristics to guarantee both good technical performance of the final products and limited environmental impact. Among the main environmental concerns are avoiding competition with food and feed crops and being climate-resilient [15]. Furthermore, there is a need to ensure the availability of alternative lignocellulosic biomass all along the supply chain to fulfill the needs of the wood industry.

The utilization of biomass obtained from perennial plant biomass presents a promising solution that has the capacity to be an alternative material [16]. Such potential has been found in various grasses, i.e., perennial grasses such as giant reed (*Arundo donax* L.) or miscanthus (*Miscanthus* × *giganteus* Greef et Deuter) [17,18]. These plants are characterized by cyclic renewability, low cultivation input requirements, resistance to environmental stresses, relatively low production costs, and high biomass yields [19]. Previous studies reported the suitability of perennial plant biomass for the manufacturing of particleboards dedicated mostly in furniture and construction purposes [16,17,20]. Another kind of non-woody plant, which might be used in board production, is dicotyledonous plants. These species have been investigated as potential sources of biomass. Besides many annual species, perennial species can also be a significant source of non-woody biomass. Jerusalem artichoke or topinambur (*Helianthus tuberosus* L.), willow-leaf sunflower (*Helianthus salicifolius* A. Dietr), wirginia mallow or fanpetals (*Sida hermaphrodita* Rusby) and cup-plant (*Silphium perfoliatum* L.) are examples of numerous species confirmed as a good source of biomass for industrial purposes [21,22,23,24].

Special attention could be paid to the cup plant due to its yield potential, multiple applications, and environmental benefits related to its long-term cultivation [22]. These perennial species are particularly valuable for non-woody biomass production, which can be used in a variety of sectors, from bio-energy and bio-products to sustainable packaging and materials. As global trends lean toward sustainable and renewable biomass sources for industry, perennial crops play a crucial role in providing raw materials without competing with food crops or overexploiting natural ecosystems [25]. Furthermore, the utilization of plant-based materials could serve as an additional crucial factor in the process of CO_2_ fixation and the promotion of sustainable construction [26,27].

Environmental changes and an increase in average temperatures are driving the development and demand for insulation boards. Well-chosen insulation materials reduce heating costs in winter and cooling costs in summer and provide a feeling of thermal comfort indoors [28]. The use of biomass as a viable replacement for traditional insulation is being suggested based on its beneficial environmental impact [29,30]. The market offers a diverse selection of insulation materials, varying in price, properties and application. A high-quality insulation material should have sufficient thermal and acoustic insulation properties, as well as fire-resistant properties [31]. These qualities are largely dependent on the kind of biomass used for the manufacturing of the panels.

There are several studies on the use of lignocellulosic materials to produce thermal insulation panels from alternative or waste materials, such as coconut shells and sugarcane bagasse [32], cotton stalk fibers, wood waste [33], aquatic weeds such as water hyacinth [34,35] and hemp [36]. However, specific studies considering the production of insulation panels with biomass derived from perennial lignocellulosic biomass are scarce in the literature [37,38,39]. However, in numerous research studies, plant biomass was used as a substitute for expanded polystyrene [40,41], not as a wood alternative. With this in mind, a research hypothesis was developed: medium-density particleboards manufactured with biomass from perennial plants show insulating properties comparable to those of a reference board manufactured from industrial wood particles.

In the case of wood-based products, in a recent study, particleboards based on miscanthus fibers were produced for insulation applications by Eschenhagen et al. [42]. According to the results, the insulation boards produced achieved promising results compared to other alternative raw materials, such as sunflower stalks. However, they were poorer than common insulation materials, such as expanded polystyrene, rock and glass walls. Klímek et al. [21] used stalks of the cup plant for particleboard production and concluded that this could be used in furniture production, meeting European Standard EN 312 type P1 [43].

Considering the potential for these species to provide high biomass yield while being cultivated on abandoned lands excluded from agricultural use. Thus, avoiding competition with food and feed crops, they could represent a pivotal resource for overcoming the current challenges of the wood industry as a source of non-wood lignocellulosic material. The main aim of this study was to examine the possibility of producing insulation boards from biomass of five types of perennial plants in comparison with boards produced from industrial wood particles.

## 2. Materials and Methods

### 2.1. Collection of Perennial Grass Biomass and Preparation of Materials

Industrial wood particles (mainly from Scots pine) were the reference material for the insulation boards in the research. Five types of perennial plant biomass were used as their substitute, namely: prairie cordgrass—(*Spartina pectinata* Link), giant miscanthus (*Miscanthus* × *giganteus* J.M. Greef & Deuter ex Hodkinson & Renvoize), switchgrass (*Panicum virgatum* L.), tall wheatgrass (*Elymus elongatus* (Host) D.R. Dewey) and cup plant or rosinweed (*Silphium perfoliatum* L.). From each perennial plant species, approximately 20 kg of air-dried biomass harvested from a growing area of ca. 200 m^2^ was obtained for testing. The biomass was harvested using an ALKO 5001 R-II drum mower (ALKO, Wittenberg, Germany) and subsequently dried to a moisture content of approximately 10%.

**Figure 1:**
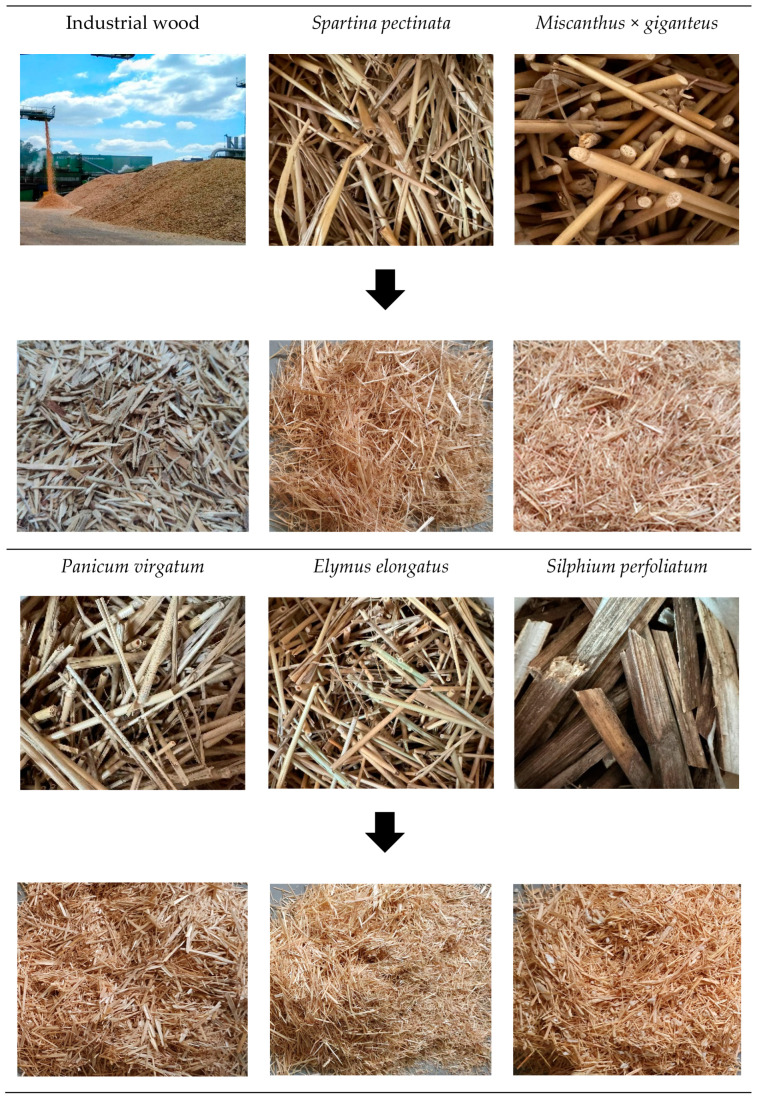
The stems of perennial plants and industrial wood particles before and after shredding.

Stems of the above-mentioned plants were crushed using a Condux mill (Mankato, MN, USA) with a knife reach of 0.9 mm. Subsequently, the useful fraction was separated and dried to the desired moisture content. The industrial wood chips and perennial plants investigated, along with the particles produced from them, are shown in Figure 1.

The next step was to determine the moisture content according to EN 322 [44] and poured bulk density (ρ) was determined according to Formula (1):

(1) $$\rho =\frac{m_{c}-m_{n}}{V}\cdot [\mathrm{kg}/m^{3}]$$

where m_c_ is the weight of the measuring vessel with the raw material [kg], m_n_ is the mass of the measuring vessel [kg], and V the capacity measuring vessel [m^3^].

The moisture content of the particles for these tests was about 7–8%, the same as during storage. Subsequently, the fractional composition was determined on an AS 200 tap laboratory vibrating screen (Fritsch, Idar-Oberstein, Germany). Mesh sieves were equipped with sieves 8.0, 4.0, 2.0, 1.0, 0.50 and 0.025 mm. The determination of the basic chemical constituents and extractives in the raw materials, such as the content of cellulose, lignin, hemicelluloses and water-soluble substances, was carried out using methods marked in TAPPI [45,46,47,48,49] and other works [10,50].

Additionally, the particles’ pH, acid and alkali buffer capacity were determined. The determination was carried out for water extracts prepared using completely dry industrial particles or biomass, cooked for 30 min at 100 °C. The extracts were titrated potentiometrically with sodium hydroxide or sulfuric acid solutions using the TitroLine Alpha Plus (SCHOTT Instruments, Mainz, Germany).

### 2.2. Production of Particleboard

The work produced reference particleboard from industrial particles and 15 board variants, i.e., 5 types of perennial plant biomass in 3 substitutions, i.e., 30, 50 and 75% of their share in the board. In total, three boards of each variant were produced. A single-level hydraulic press (Simpelkamp, Krefeld, Germany) was used to produce single-layer boards with dimensions of 700 × 500 × 50 mm and a nominal density of 250 kg/m^3^. The actual density of manufactured boards varied from 220 kg/m^3^ to 242 kg/m^3^ (Table 1). For production, the following pressing parameters were used: a unit pressure of 2.5 MPa, temperature of 180 °C and pressing ratio of 15 s per mm of nominal board thickness. The boards were conditioned in a conditioning chamber at a relative humidity of 65 ± 5% and a temperature of 20 ± 2 °C. Melamine–urea–formaldehyde (MUF) adhesive (Swiss Krono Sp. z o.o., Zary, Poland) was used to glue the particles, and the hardener was a 40% water solution composed of NH_4_NO_3_. Resinification was set at 10% of dry particles (*w*/*w*), calculated using a 3.0 wt.% NH_4_NO_3_ hardener. The solid content of the adhesive mass was 68.4%, the pH was 7.95 and the dynamic viscosity was 254 mPa∙s.

The following determinations were made for the boards: density was measured according to EN 323 [51] and the internal bond (IB) was measured according to EN 319 [52] (eight samples per board), and formaldehyde emissions were measured using the chamber method according to ASTM D6007-14 [53] (nine samples per variant). Samples were conditioned before research for 168 ± 3 h at 24 ± 3 °C and at a relative humidity of 50 ± 5%. In addition, the values of the specific parameters of thermal insulation properties, namely the heat transfer coefficient (U), which was the direct result of research, and the thermal conductivity coefficient (λ), were also calculated. Measurements of thermal resistance were performed using a Linseis HFM 300 (Selb, Germany) instrument IRMM-440A. Measurements were carried out for temperatures of 20 °C and 0 °C, according to the standard EN 12667 [54] with four repetitions per board.

## 3. Results and Discussion

Chemical composition and cell wall structure are two key characteristics of lignocellulosic raw materials [55]. By shaping the geometry of the fiber, the cell wall structure plays an important role in its morphological properties, such as elasticity and strength. The chemical composition, on the other hand, directly determines the physical and mechanical properties of the raw material, including hardness and thermal stability and hydrophobicity [24]. This means that the type of raw material results from both the biological origin and the specific chemical composition. Moreover, it significantly affects the quality and suitability of the resulting materials, such as particles and, subsequently, particleboards from perennial plants.

**Figure 2:**
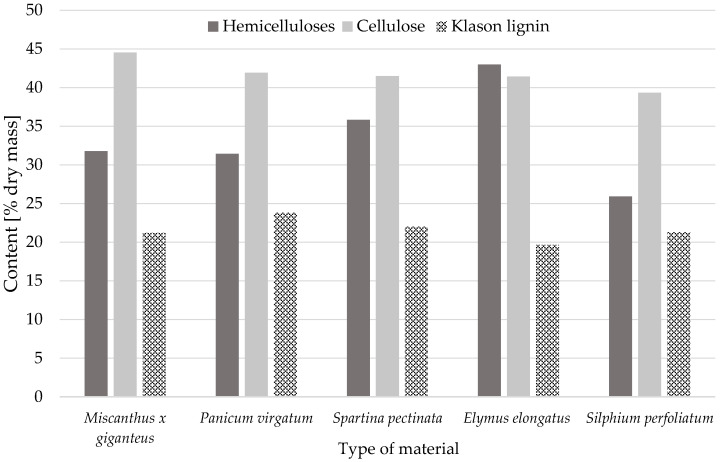
Chemical composition of tested perennial plants.

**Figure 3:**
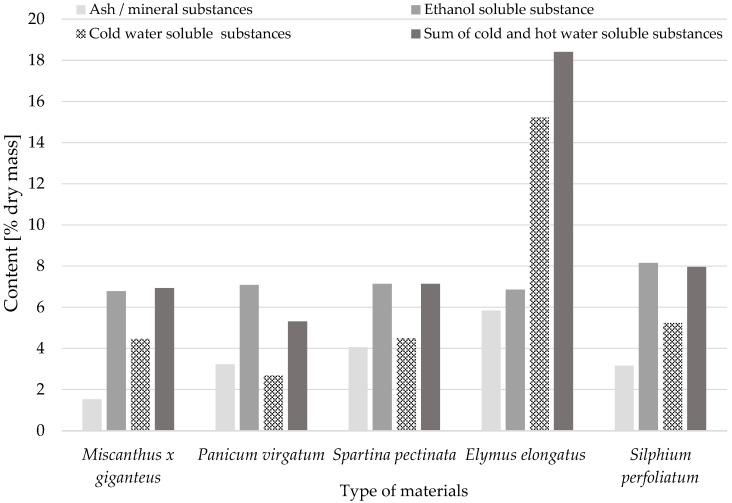
Content of mineral substances (ash) and extractives (solvent: ethanol, cold water, cold and hot water together) in tested perennial plants.

A detailed analysis of the chemical composition and cell wall structure enables a better understanding of the potential of these raw materials in various industrial applications and indicates the possible modifications and optimisations of the production process to obtain the desired functional properties. This information has important implications for further performance properties. It will determine whether the processing of the perennial grass straws used in the research could be a future direction for the use of this raw material in the wood-based panel industry in the construction sector. Figure 2 and Figure 3 show the chemical composition and content of chemical components for the perennial plants researched.

The moisture content of the samples after grinding ranged from 7.65% to 9.22%. Each of the analyzed material was characterized by almost 40% cellulose content, the lowest content was noticed in the case of rosinweed i.e., 39.32%, and the highest level of cellulose content, 44.53%, was observed in the giant miscanthus samples. The lignin content was generally lower than cellulose, ranging from 19.65% to 23.81% (Figure 2). The content of mineral substantion (ash) was very diverse between examined plants. The lowest value was 1.53% (giant miscanthus), while the highest was 5.84% (wheatgrass) of dried mass. The water extract summed in cold and hot water in the raw materials was around 5–8% of dry weight, the exception was the sample of tall wheatgrass, which contained as much as 18% extract with dry weight. The sample was previously ethanol-extracted (96%) before the determination of cellulose and lignin content. The content of the ethanol extract ranged from 1.89% (rosinweed) to 8.09% (tall wheatgrass). A substantial portion of the sample demonstrated solubility in a 1% NaOH solution, with identified compounds ranging from 25.91% (rosinweed) to 42.99% (tall wheatgrass) (Figure 3).

**Figure 4:**
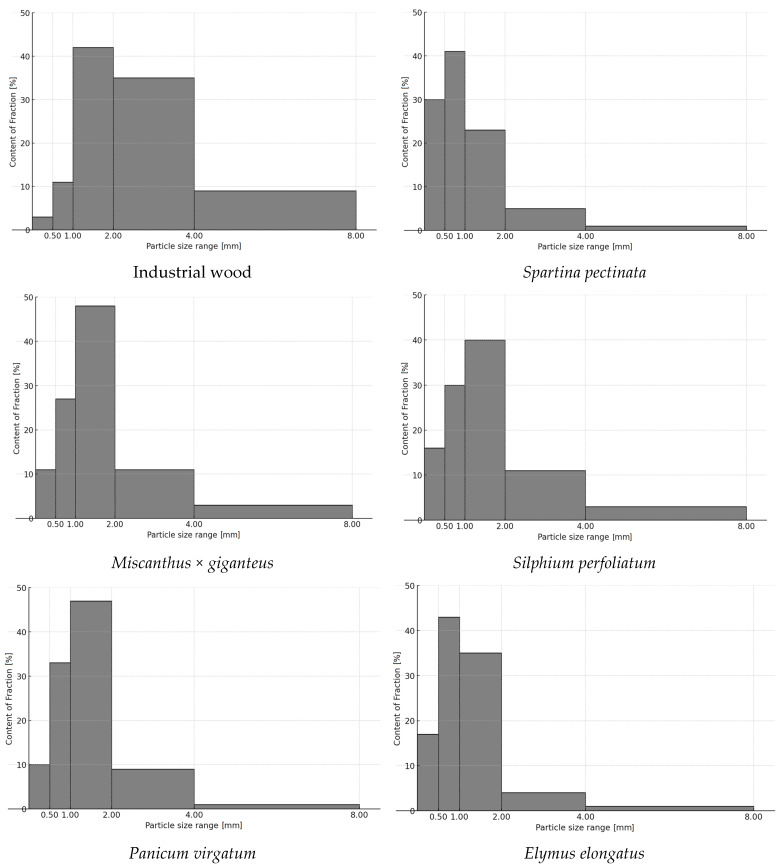
Fractional composition of industrial wood particles and particles of five types of perennial plant biomass.

Figure 4 shows the fractional composition of industrial wood and perennial plant particles.

The analysis shows that the sieve fractions 2.00–4.00 mm and 4.00–8.00 mm represent a low percentage of perennial plant particles compared to industrial particles, for which they represent about 35 and 9%, respectively. Tall wheatgrass and prairie cordgrass have the lowest proportion of these fractions at 5%, while giant miscanthus and cup plants have the highest at 14%. Switchgrass falls in the middle with a proportion of 10%. The results indicate that particles with a size of 0.50 and 1.00 mm represent the largest mass share for the tested plant biomass. For particles from prairie cordgrass and tall wheatgrass, the largest fractions were from the 0.5 mm sieve and were approximately 41% and 43%, respectively. For wood industrial particles and cup plants, the highest fractions were 42% and 40%, and for giant miscanthus and switchgrass, it was about 48% for a particle size of 1.00 mm sieve. In terms of the particles that passed through the 0.50 mm sieve, by far the largest share of around 30% was derived from prairie cordgrass. For tall wheatgrass and cup plants, the proportion of fine particles is approximately 17%. In the cases of giant miscanthus and switchgrass, the proportion is significantly less, approx. 10%, and it is only 3% for woody industrial particles.

**Figure 5:**
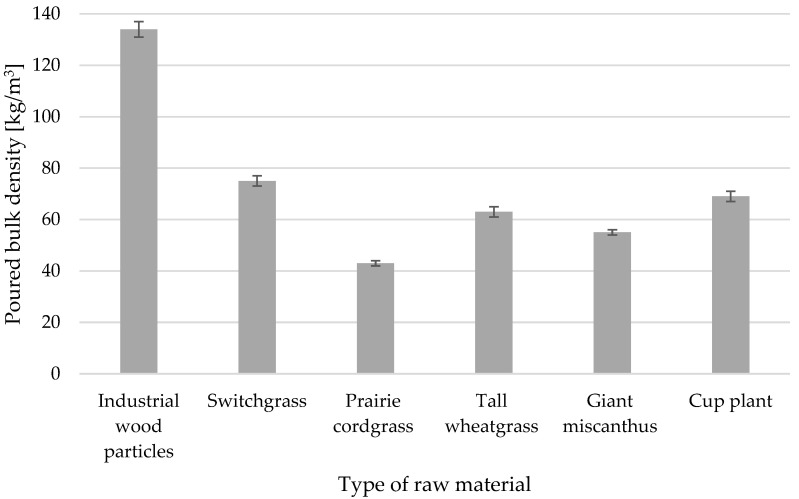
The poured bulk density of the industrial wood and perennial plant particles.

Figure 5 shows the poured bulk density of the particles. The bulk density of tested perennial plant biomass is between 40 and 75 kg/m^3^. A value in this range, i.e., about 49 kg/m^3^ for a similar moisture content (approx. 8%) is given for prairie grass [56], while for rapeseed straw, with a moisture content of approximately 3%, the density is about 41 kg/m^3^ [57]. For wood industrial particles, the value was similar to that for industrial particles for the core layer in particleboards (approx. 141 kg/m^3^) researched by Neitzel et al. [58], i.e., approx. 134 kg/m^3^.

The buffering capacity of the lignocellulosic material affects the polymerization quality and curing rate of urea–formaldehyde (UF) resins [59]. As a result, the mechanical properties of the boards made from them are affected by curing and bond formation with the adhesive system [58]. Research reported that substances extracted from different wood species had a significant effect on the curing and adhesion of petroleum-based synthetic resins [60]. The pH values of the examined raw materials ranged between 4.5 and 6.0; however, all tested samples except prairie cordgrass have a pH of between 4.5 and 5.0 (Table 2). Prairie cordgrass was characterized by the highest pH, i.e., 6.0, and the lowest pH was observed for industrial wood. When it comes to buffer capacity, acid- and alkaline-type buffers were measured. The highest acid buffer capacity was measured in tall wheatgrass, while the lowest was in prairie cordgrass. In the case of alkaline buffer capacity, the highest value was measured in industrial raw material, while the lowest was found in switchgrass. The alkaline buffer capacity was highest among the raw materials researched was 5.63 mmol H_2_SO_4_/100 g for industrial wood at a pH of 4.5. The lowest amount of acid was found for switchgrass and giant miscanthus at measures below 2 mmol H_2_SO_4_/100 g. This is comparable with a capacity of 10.03 mEq for non-resinized fresh wood particles at a pH of 5.19 [61].

Extremely low or high pH values negatively affect the bonding of lignocellulosic particles and the bonding agent, potentially resulting in reduced mechanical strength [62]. For example, a material with a significantly low pH can affect the gel time of urea–formaldehyde adhesives, which can eliminate their use due to insufficient time for matting and pressing [63]. Formaldehyde emissions in particleboards are shown in Table 3, taking into account the type of perennial plant and the degree of substitution in the particleboard.

In terms of formaldehyde emissions, all boards met the ASTM D6007-14 standard, indicating 0.1 ppm as the maximum value. A value of 0.05 ppm was achieved in boards of all grass types at 30% and in boards of 75% switchgrass and giant miscanthus, as well as in boards of 50% prairie cordgrass and switchgrass.

**Figure 6:**
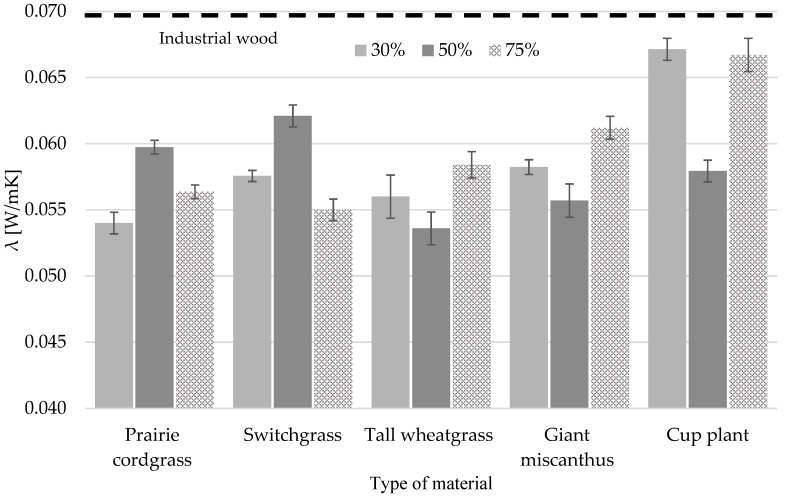
The effect of the proportion of different types of perennial plants on the value of the thermal conductivity coefficient.

The crucial factors influencing the thermal properties of wood insulation materials are density, temperature and the thickness effect of moisture [64,65,66]. For insulation, lower density leads to better thermal insulation, i.e., lower thermal conductivity. In contrast, a thicker material also increases thermal resistance, leading to a lower heat transfer coefficient. Figure 6 shows the effect of the proportion of different types of perennial grass on the thermal conductivity coefficient λ. The lowest λ value of 0.054 W/mK was shown to be 50% tall wheatgrass and 30% prairie cordgrass. Of the highest percentage of grass in the particleboards, switchgrass showed the lowest parameter and was 0.055 W/mK. Cup plant particleboards have the worst insulating properties. Materials with a low λ are more effective in preventing heat flow through the building envelope. Striving for the lowest possible value for these coefficients is crucial in the design and construction of energy-efficient buildings. It is noteworthy, however, that each board variant containing perennial grass showed better insulating properties than particleboard made from industrial wood particles. In the best-performing variant, this was a decrease of more than 22% (0.016 W/mK).

**Figure 7:**
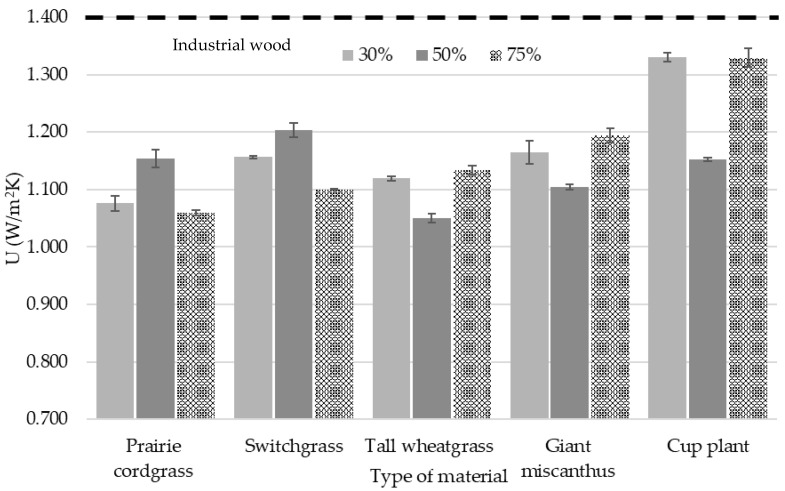
Effect of the proportion of different perennial plants on the value of the heat transfer coefficient.

The same observations were made for the heat transfer coefficient—U (Figure 7). Apart from the boards with 30 and 75% of cup plant and the board with industrial wood particles, the U-value was below 1.2 W/m^2^K for each board. The lowest U value was exhibited by 50% tall wheatgrass and prairie cordgrass at 75 and 30%. All the composites produced from perennial grasses showed competitive insulation features in comparison to other bio-based insulating materials, such as wood fibers/textile waste insulation boards (0.078–0.089 W/mK) [67], rapeseed straw concrete (0.094 W/mK) [68] and corn cob (0.13–0.15 W/mK) [69]. These exhibited thermal conductivity values close to commercially available bio-sourced insulation products such as hemp insulation (0.038–0.043 W/mK) [70] and non-renewable insulation products such as rock wool (0.036 W/mK) and expanded polystyrene (0.038 W/mK) [71]. Furthermore, the obtained values are in line with the very promising insulating boards produced from the rice straw presented in the recent study by Zhou et al. [72]. Insulating blocks were produced from coriander straw (approx. 53% cellulose and 21% hemicelluloses), in which the λ at a density of approx. 190 kg/m^3^ was 0.067 W/mK; while at 125 kg/m^3^, the density was approx. 0.057 W/mK [73]. The particleboards produced by Eschenhagen et al. [42] with miscanthus fibers showed values of thermal conductivity ranged from 0.057 to 0.068 W/mK, which was slightly higher than ours of the same species. According to EN 13986, the thermal conductivity of particleboards with an average density of 300 kg/m^3^ should be 0.07 W/mK [74].

**Figure 8:**
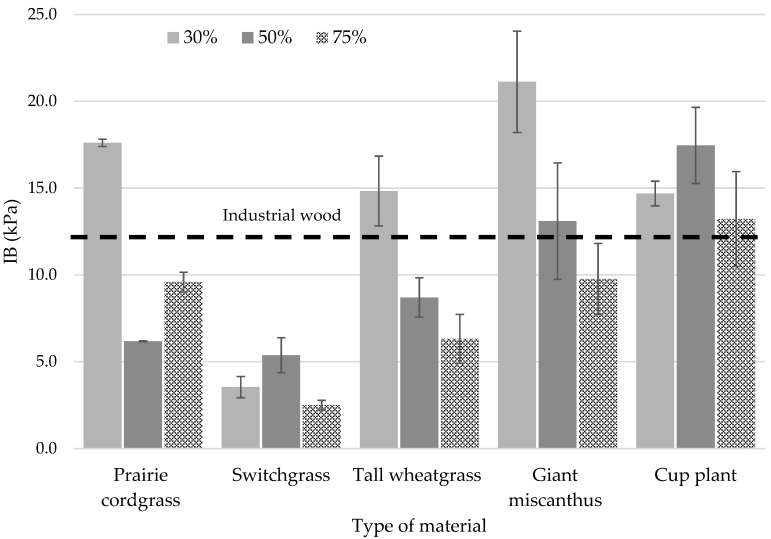
Effect of the proportion of different perennial plants on the value of the internal bond.

Higher IB values were shown for particleboards with a lower grass content, in the range of 14.7–21.1 kPa (Figure 8). For four of the five grass types examined, the highest IB values were for boards with a 30% share. Only the board with the prairie cordgrass type showed very low properties over the entire share range, obtaining a maximum of 5.4 kPa. Relative to the board with industrial particles, this is more than a twofold decrease in IB values. As a grass type, it was the cup plant that showed the highest IB, ranging from 13.2 to 17.5 kPa for the variant with a 50% proportion. In this research, the boards had a nominal density of 250 kg/m^3^ and a maximum IB value of 21.1 kPa. These values do not meet and are far from the minimum requirements for particleboards in EN 312 [43].

Considering the results of the research, the hypothesis was partially fulfilled. The raw materials researched have the potential to produce particleboards with thermal properties similar to those of wood particles. The thermal conductivity value obtained was less than 0.07 W/mK (0.054–0.067 W/mK) in each variant of perennial plants. The highest thermal conductivity values were recorded for the cup plant content at 30 and 75% in boards (>0.065 W/mK), but at a 50% proportion, the value was only 0.057 W/mK. In this respect, switchgrass (0.055 W/mK) and prairie cordgrass (0.056 W/mK) performed the best for perennial plants in boards, i.e., at a 75% share. In contrast, for a wood substitution of 50% perennial plants, tall wheatgrass and giant miscanthus were the most favorable (0.054 and 0.056 W/mK, respectively). At 30% perennial grass in a board, the best result (0.054 W/mK) was obtained with prairie cordgrass. Still taking into account the formaldehyde emissions from the boards, the most favorable options are switchgrass at 75% (0.053 ppm), giant miscanthus at 50% (0.042 ppm) and prairie cordgrass at 30% (0.050 ppm). To address limitations, future studies should increase the strength properties, including internal bond. According to the literature, not every raw material can be used for the production of particleboards and parameters should be analyzed to confirm suitability for thermal insulation applications. To achieve these results, it is necessary to analyze the impact of smaller proportions of alternative biomass to improve strength. In addition, tests should continue to check the impact parameters, such as density, pressing parameters and adhesives. It may also be useful to use computer modeling tools designed with assumed parameters to simulate the distribution of forces in slabs with different proportions of plants.

## 4. Conclusions

With global trends towards sustainable and renewable sources of raw materials, perennial plants offer a promising alternative to conventional insulation materials. Their ability to grow on marginal land means that they do not compete with food crops, and combined with their low fertilizer requirements, they are a favorable choice from an environmental perspective. As the research results show, the potential to produce insulation board using perennial plants has been confirmed. Each of the five plant species tested could be used as raw material for board production (thermal conductivity in the range 0.054–0.067 W/mK). However, the results did not completely confirm the hypothesis, mainly due to the low mechanical properties of the boards, such as internal bond. However, the positive effect of the presence of perennial plants in the boards was noted for formaldehyde emissions. The results of formaldehyde emission tests performed on manufactured boards show that, in most cases, the type of raw material used is more important than the amount, especially for switchgrass (0.048–0.053 ppm) and giant miscanthus (0.042–0.054 ppm).

Given these advantages, further research into the use of perennial plants in the insulation industry, particularly in terms of improving internal bonds, could contribute to the development of more sustainable building technologies and reduce the exploitation of traditional resources. Given the intensification of efforts to prepare lignocellulosic biomass panel materials with sufficient technical parameters for use in wooden frame construction, many variants of both process variables and raw materials should be studied. In the long term, it may be beneficial to combine different raw materials within a single particleboard to form combinations.

## Figures and Tables

**Table 1:** The actual particleboard density according to variants [kg/m^3^].

Type of Raw Material	Share [%]	Board Desnity [kg/m^3^]
Industrial wood	100	239 ± 1
Prairie cordgrass	30	220 ± 2
	50	226 ± 3
	75	221 ± 3
Switchgrass	30	239 ± 3
	50	229 ± 3
	75	236 ± 2
Tall wheatgrass	30	233 ± 1
	50	227 ± 2
	75	220 ± 2
Giant miscanthus	30	239 ± 3
	50	238 ± 5
	75	229 ± 1
Cup plant	30	237 ± 1
	50	234 ± 1
	75	242 ± 2

**Table 2:** Values of measured pH and buffer capacity of tested raw materials.

Property	Type of Raw Material					
	Industrial Wood	Switchgrass	Prairie Cordgrass	Tall Wheatgrass	Giant Miscanthus	Cup Plant
pH	4.5 ± 0.1	4.9 ± 0.1	6.0 ± 0.2	5.0 ± 0.1	4.7 ± 0.1	5.0 ± 0.0
Acid buffer capacity [mmol NaOH/100 g]	2.53 ± 0.04	2.38 ± 0.07	1.97 ± 0.10	5.61 ± 0.06	2.93 ± 0.03	4.8 ± 0.06
Alkaline buffer capacity [mmol H_2_SO_4_/100 g]	5.63 ± 0.06	1.85 ± 0.10	4.23 ± 0.03	3.72 ± 0.01	1.89 ± 0.04	4.83 ± 0.07

**Table 3:** The hygienic properties of manufactured particleboards.

Type of Raw Material	Share [%]	FE [ppm]
Industrial wood	100	0.100
Prairie cordgrass	30	0.050
	50	0.052
	75	0.079
Switchgrass	30	0.052
	50	0.048
	75	0.053
Tall wheatgrass	30	0.052
	50	0.058
	75	0.059
Giant miscanthus	30	0.054
	50	0.042
	75	0.045
Cup plant	30	0.054
	50	0.070
	75	0.080

## Data Availability

Data are contained within the article.

## References

[1] FAO (2020). Global Forest Resources Assessment 2020.

[2] Köhl M., Linser S., Prins K., Talarczyk A. (2021). The EU Climate Package “Fit for 55”—A Double-Edged Sword for Europeans and Their Forests and Timber Industry. For. Policy Econ..

[3] De Lombaerde E., Vangansbeke P., Lenoir J., Van Meerbeek K., Lembrechts J., Rodríguez-Sánchez F., Luoto M., Scheffers B., Haesen S., Aalto J. (2022). Maintaining Forest Cover to Enhance Temperature Buffering under Future Climate Change. Sci. Total Environ..

[4] Reyer C., Guericke M., Ibisch P.L. (2009). Climate Change Mitigation via Afforestation, Reforestation and Deforestation Avoidance: And What about Adaptation to Environmental Change?. New For..

[5] Green M., Taggart J. (2020). Tall Wood Buildings: Design, Construction and Performance.

[6] Pajchrowski G., Noskowiak A., Lewandowska A., Strykowski W. (2014). Wood as a Building Material in the Light of Environmental Assessment of Full Life Cycle of Four Buildings. Constr. Build. Mater..

[7] Michalec K., Wąsik R., Gach M. (2023). Impact of the Presence of Foreign Bodies on Quality and Value of Oak Timber. Drewno. Pr. Nauk. Doniesienia Komun. Wood. Res. Pap. Rep. Announc..

[8] Łukawski D., Hochmańska-Kaniewska P., Janiszewska-Latterini D., Lekawa-Raus A. (2023). Functional Materials Based on Wood, Carbon Nanotubes, and Graphene: Manufacturing, Applications, and Green Perspectives. Wood Sci. Technol..

[9] Ahmed M.Z., Sikora K.S. (2024). Mechanical Properties of Cross Laminated Panels Made From Date Palm Fibres. Drewno. Pr. Nauk. Doniesienia Komun. Wood. Res. Pap. Rep. Announc..

[10] Tomczak K., Mania P., Cukor J., Vacek Z., Tomczak A. (2024). Wood Quality of Pendulate Oak on Post-Agricultural Land: A Case Study Based on Physico-Mechanical and Anatomical Properties. Forests.

[11] Zeidler A., Borůvka V., Tomczak K., Vacek Z., Cukor J., Vacek S., Tomczak A. (2024). The Potential of Non-Native Pines for Timber Production—A Case Study from Afforested Post-Mining Sites. Forests.

[12] Pędzik M., Tomczak K., Janiszewska-Latterini D., Tomczak A., Rogoziński T. (2022). Management of Forest Residues as a Raw Material for the Production of Particleboards. Forests.

[13] Pędzik M., Janiszewska D., Rogoziński T. (2021). Alternative Lignocellulosic Raw Materials in Particleboard Production: A Review. Ind. Crops Prod..

[14] Pędzik M., Júda M., Kminiak R., Czerniejewska-Wolska H., Rogoziński T. (2024). The Effect of Average Chip Thickness on the Potentially Respirable Dust from CNC Finish Milling of Wood-Based Materials. Drewno. Pr. Nauk. Doniesienia Komun. Wood. Res. Pap. Rep. Announc..

[15] Rathour R.K., Behl M., Dhashmana K., Sakhuja D., Ghai H., Sharma N., Meena K.R., Bhatt A.K., Bhatia R.K. (2023). Non-Food Crops Derived Lignocellulose Biorefinery for Sustainable Production of Biomaterials, Biochemicals and Bioenergy: A Review on Trends and Techniques. Ind. Crops Prod..

[16] Janiszewska D., Żurek G., Martyniak D., Bałęczny W. (2022). Lignocellulosic Biomass of C3 and C4 Perennial Grasses as a Valuable Feedstock for Particleboard Manufacture. Materials.

[17] Klímek P., Wimmer R., Meinlschmidt P., Kúdela J. (2018). Utilizing Miscanthus Stalks as Raw Material for Particleboards. Ind. Crops Prod..

[18] Nassi o di Nasso N., Roncucci N., Triana Jimeno F., Tozzini C., Bonari E. (2011). Productivity of Giant Reed (*Arundo Donax* L.) and Miscanthus (*Miscanthus* × *Giganteus* Greef et Deuter) as Energy Crops: Growth Analysis. Ital. J. Agron..

[19] Scordia D., Cosentino S. (2019). Perennial Energy Grasses: Resilient Crops in a Changing European Agriculture. Agriculture.

[20] Trischler J., Sandberg D. (2014). Monocotyledons in Particleboard Production: Adhesives, Additives, and Surface Modification of Reed Canary Grass. Bioresources.

[21] Klímek P., Meinlschmidt P., Wimmer R., Plinke B., Schirp A. (2016). Using Sunflower (Helianthus Annuus L.), Topinambour (*Helianthus tuberosus* L.) and Cup-Plant (*Silphium perfoliatum* L.) Stalks as Alternative Raw Materials for Particleboards. Ind. Crops Prod..

[22] Cumplido-Marin L., Graves A.R., Burgess P.J., Morhart C., Paris P., Jablonowski N.D., Facciotto G., Bury M., Martens R., Nahm M. (2020). Two Novel Energy Crops: *Sida hermaphrodita* (L.) Rusby and *Silphium perfoliatum* L.—State of Knowledge. Agronomy.

[23] Peni D., Stolarski M.J., Dębowski M. (2022). Green Biomass Quality of Perennial Herbaceous Crops Depending on the Species, Type and Level of Fertilization. Ind. Crops Prod..

[24] Shadhin M., Rahman M., Jayaraman R., Chen Y., Mann D., Zhong W. (2023). Natural Biomass &amp; Waste Biomass Fibers—Structures, Environmental Footprints, Sustainability, Degumming Methods, &amp; Surface Modifications. Ind. Crops Prod..

[25] Kowalska G., Baj T., Kowalski R., Hanif M.A. (2022). Characteristics of Selected Silphium Species as Alternative Plants for Cultivation and Industry with Particular Emphasis on Research Conducted in Poland: A Review. Sustainability.

[26] Bozsaky D. (2019). Nature-Based Thermal Insulation Materials From Renewable Resources—A State-Of-The-Art Review. Slovak. J. Civ. Eng..

[27] Ljungberg L.Y. (2007). Materials Selection and Design for Development of Sustainable Products. Mater. Des..

[28] Fu H., Ding Y., Li M., Li H., Huang X., Wang Z. (2020). Research on Thermal Performance and Hygrothermal Behavior of Timber-Framed Walls with Different External Insulation Layer: Insulation Cork Board and Anti-Corrosion Pine Plate. J. Build. Eng..

[29] Latif E., Ciupala M.A., Tucker S., Wijeyesekera D.C., Newport D.J. (2015). Hygrothermal Performance of Wood-Hemp Insulation in Timber Frame Wall Panels with and without a Vapour Barrier. Build. Environ..

[30] Rojas C., Cea M., Iriarte A., Valdés G., Navia R., Cárdenas R.J.P. (2019). Thermal Insulation Materials Based on Agricultural Residual Wheat Straw and Corn Husk Biomass, for Application in Sustainable Buildings. Sustain. Mater. Technol..

[31] Berge A., Johansson P. (2012). Literature Review of High Performance Thermal Insulation.

[32] Panyakaew S., Fotios S. (2011). New Thermal Insulation Boards Made from Coconut Husk and Bagasse. Energy Build..

[33] Cetiner I., Shea A.D. (2018). Wood Waste as an Alternative Thermal Insulation for Buildings. Energy Build..

[34] Philip S., Rakendu R. (2020). Thermal Insulation Materials Based on Water Hyacinth for Application in Sustainable Buildings. Mater. Today Proc..

[35] Salas-Ruiz A., del Mar Barbero-Barrera M., Ruiz-Téllez T. (2019). Microstructural and Thermo-Physical Characterization of a Water Hyacinth Petiole for Thermal Insulation Particle Board Manufacture. Materials.

[36] Charai M., Sghiouri H., Mezrhab A., Karkri M. (2021). Thermal Insulation Potential of Non-Industrial Hemp (Moroccan Cannabis Sativa L.) Fibers for Green Plaster-Based Building Materials. J. Clean. Prod..

[37] Krenn S., Huber H., Barbu M.C., Petutschnigg A., Schnabel T. (2017). Insulation Boards Made of Annual and Perennial Plants Bonded with Tannins and Other Adhesives. Pro Ligno.

[38] Nagl L., Barbu M.C., Schnabel T., Petutschnigg A., Jäger A., Huber H. (2015). Use of Annual and Perennial Plants for Dimensionally Stable Insulation Panels. Pro Ligno.

[39] Gaspar F., Bakatovich A., Davydenko N., Joshi A. (2020). Building Insulation Materials Based on Agricultural Wastes. Bio-Based Materials and Biotechnologies for Eco-Efficient Construction.

[40] Moll L., Höller M., Hubert C., Korte C.A.C., Völkering G., Wever C., Pude R. (2022). Cup Plant (*Silphium perfoliatum* L.) Biomass as Substitute for Expanded Polystyrene in Bonded Leveling Compounds. Agronomy.

[41] Schulte M., Lewandowski I., Pude R., Wagner M. (2021). Comparative Life Cycle Assessment of Bio-based Insulation Materials: Environmental and Economic Performances. GCB Bioenergy.

[42] Eschenhagen A., Raj M., Rodrigo N., Zamora A., Labonne L., Evon P., Welemane H. (2019). Investigation of Miscanthus and Sunflower Stalk Fiber-Reinforced Composites for Insulation Applications. Adv. Civ. Eng..

[43] (2010). Particleboards–Specifications.

[44] (1993). Wood-Based Panels–Determination of Moisture Content.

[45] TAPPI (1996). Test Methods (1996–1997).

[46] TAPPI (2007). T 204 cm-97 Solvent Extractives of Wood and Pulp.

[47] TAPPI (2008). T 207 CM Water Solubility of Wood and Pulp.

[48] TAPPI (2006). T 222 om-02 Acid-Insoluble Lignin in Wood and Pulp.

[49] TAPPI (2012). T 212 OM-12 1% Sodium Hydroxide Solubility of Wood and Pulp.

[50] Kulak P., Komorowicz M., Lachowicz H. (2024). Fibre Morphological Characteristics, Chemical Composition and Fuel Properties of Paulownia COTEVISA-2 Wood. Eur. J. Wood Wood Prod..

[51] (1993). Wood-Based Panels—Determination of Density. European Committee for Standardization.

[52] (1993). Particleboards and Fibreboards—Determination of Tensile Strength Perpendicular to the Plane of the Board.

[53] (2014). Standard Test Method for Determining Formaldehyde Concentrations in Air from Wood Products Using a Small-Scale Chamber.

[54] (2001). Thermal Performance of Building Materials and Products—Determination of Thermal Resistance by Means of Guarded Hot Plate and Heat Flow Meter Methods—Products of High and Medium Thermal Resistance.

[55] Karimah A., Ridho M.R., Munawar S.S., Adi D.S., Damayanti R., Subiyanto B., Fatriasari W., Fudholi A. (2021). A Review on Natural Fibers for Development of Eco-Friendly Bio-Composite: Characteristics, and Utilizations. J. Mater. Res. Technol..

[56] Lam P.S., Sokhansanj S., Bi X., Lim C.J., Naimi L.J., Hoque M., Mani S., Womac A.R., Narayan S., Ye X.P. (2008). Bulk Density of Wet and Dry Wheat Straw and Switchgrass Particles. Appl. Eng. Agric..

[57] Dukarska D., Pędzik M., Rogozińska W., Rogoziński T., Czarnecki R. (2021). Characteristics of Straw Particles of Selected Grain Species Purposed for the Production of Lignocellulose Particleboards. Part. Sci. Technol..

[58] Neitzel N., Eder M., Hosseinpourpia R., Walther T., Adamopoulos S. (2023). Chemical Composition, Particle Geometry, and Micro-Mechanical Strength of Barley Husks, Oat Husks, and Wheat Bran as Alternative Raw Materials for Particleboards. Mater. Today Commun..

[59] Andre N., Young T.M., Rials T.G. (2006). On-Line Monitoring of the Buffer Capacity of Particleboard Furnish by Near-Infrared Spectroscopy. Appl. Spectrosc..

[60] Liu Z., Mi Y., Kan Y., Bai Y., Li J., Gao Z. (2023). Evaluation of the Interactions of Typical Wood Extracts on the Bonding Performance of Soybean-Based Adhesives. Polym. Test..

[61] Wan H., Wang X.-M., Jun S. (2014). Recycling Wood Composite Panels: Characterizing Recycled Materials. Bioresources.

[62] Król P., Toczyłowska-Mamińska R., Mamiński M. (2017). A Critical Role for the Presence of Lignocellulosic Material in the Determination of Wood Buffering Capacity. J. Wood Chem. Technol..

[63] Xing C., Zhang S.Y., Deng J., Wang S. (2007). Urea–Formaldehyde-resin Gel Time as Affected by the PH Value, Solid Content, and Catalyst. J. Appl. Polym. Sci..

[64] Gusyachkin A.M., Sabitov L.S., Khakimova A.M., Hayrullin A.R. (2019). Effects of Moisture Content on Thermal Conductivity of Thermal Insulation Materials. IOP Conf. Ser. Mater. Sci. Eng..

[65] Hung Anh L.D., Pásztory Z. (2021). An Overview of Factors Influencing Thermal Conductivity of Building Insulation Materials. J. Build. Eng..

[66] Kumar D., Alam M., Zou P.X.W., Sanjayan J.G., Memon R.A. (2020). Comparative Analysis of Building Insulation Material Properties and Performance. Renew. Sustain. Energy Rev..

[67] Lacoste C., El Hage R., Bergeret A., Corn S., Lacroix P. (2018). Sodium Alginate Adhesives as Binders in Wood Fibers/Textile Waste Fibers Biocomposites for Building Insulation. Carbohydr. Polym..

[68] Rahim M., Douzane O., Tran Le A.D., Langlet T. (2016). Effect of Moisture and Temperature on Thermal Properties of Three Bio-Based Materials. Constr. Build. Mater..

[69] Viel M., Collet F., Lanos C. (2019). Development and Characterization of Thermal Insulation Materials from Renewable Resources. Constr. Build. Mater..

[70] Latif E., Tucker S., Ciupala M.A., Wijeyesekera D.C., Newport D. (2014). Hygric Properties of Hemp Bio-Insulations with Differing Compositions. Constr. Build. Mater..

[71] Ducoulombier L., Lafhaj Z. (2017). Comparative Study of Hygrothermal Properties of Five Thermal Insulation Materials. Case Stud. Therm. Eng..

[72] Zhou Y., Trabelsi A., El Mankibi M. (2023). Hygrothermal Properties of Insulation Materials from Rice Straw and Natural Binders for Buildings. Constr. Build. Mater..

[73] Uitterhaegen E., Labonne L., Ballas S., Véronèse T., Evon P. The Coriander Straw, an Original Agricultural by-Product for the Production of Building Insulation Materials. Proceedings of the 3rd International Conference on Bio-Based Building Materials (ICBBM).

[74] (2015). Wood-Based Panels for Use in Construction. Characteristics, Evaluation of Conformity and Marking.

